# Comprehensive evaluation of T7 promoter for enhanced yield and quality in mRNA production

**DOI:** 10.1038/s41598-024-59978-5

**Published:** 2024-04-26

**Authors:** Yustika Sari, Sara Sousa Rosa, Jack Jeffries, Marco P. C. Marques

**Affiliations:** 1 Department of Biochemical Engineering, University College London, Gordon Street, London, WC1E 6BT UK; 2 Department of Bioengineering, iBB—Institute for Bioengineering and Biosciences, Instituto Superior Técnico, Universidade de Lisboa, Lisbon, Portugal; 3 Associate Laboratory i4HB—Institute for Health and Bioeconomy, Instituto Superior Técnico, Universidade de Lisboa, Lisbon, Portugal

**Keywords:** Nucleic-acid therapeutics, Synthetic biology, RNA

## Abstract

The manufacturing of mRNA vaccines relies on cell-free based systems that are easily scalable and flexible compared with the traditional vaccine manufacturing processes. Typically, standard processes yield 2 to 5 g L^−1^ of mRNA, with recent process optimisations increasing yields to 12 g L^−1^. However, increasing yields can lead to an increase in the production of unwanted by-products, namely dsRNA. It is therefore imperative to reduce dsRNA to residual levels in order to avoid intensive purification steps, enabling cost-effective manufacturing processes. In this work, we exploit sequence modifications downstream of the T7 RNA polymerase promoter to increase mRNA yields whilst simultaneously minimising dsRNA. In particular, transcription performance was optimised by modifying the sequence downstream of the T7 promoter with additional AT-rich sequences. We have identified variants that were able to produce higher amounts of mRNA (up to 14 g L^−1^) in 45 min of reaction. These variants exhibited up to a 30% reduction in dsRNA byproduct levels compared to a wildtype T7 promoter, and have similar EGFP protein expression. The results show that optimising the non-coding regions can have an impact on mRNA production yields and quality, reducing overall manufacturing costs.

## Introduction

Vaccines are pivotal in mitigating severe health complications and hold the capacity to control the spread of infectious diseases, potentially eradicating these in populations^1,2^. Over the long term, the savings in healthcare expenses and reduction in mortality rates can prove economically advantageous^1,3,4^. After years of dedicated research on vaccine technology, the mRNA vaccine received full approval in 2020 to tackle COVID-19 pandemic^5,6^ with the BNT162b2 mRNA vaccine (Pfizer-BioNTech), achieved WHO emergency use authorization less than a year after the official pandemic declaration^7–9^. Between the date of approval and November 2023, approximately 1.18 × 10^12^ doses of Pfizer-BioNTech vaccines, and 3.27 × 10^11^ of the Moderna vaccines were administrated worldwide^10^. With the remarkable success against SARS-CoV-2, research in mRNA vaccines is expanding and intensifying against other infectious diseases, such as Zika, HIV^11,12^, and a spectrum of cancers^13^. Despite this, the cost of these vaccines is still prohibitive for LMICs, mainly driven by the cost of goods^14^.

The prominent feature of mRNA vaccines is their rapid manufacturing which can be swiftly developed using a pathogen’s gene sequences^15^. The mRNA vaccine is designed to mimic eukaryotic mRNA, and is composed of a 5’ cap, an open reading frame (ORF) encoding a specific antigen, untranslated regions (5’UTR and 3’UTR), and a poly(A) tail^16,17^. Inherently, these vaccines are precise and simple, since after administration only the antigen encoded by the targeted gene^18^ is translated. Furthermore, their safety profile is high since the mRNA administered has a transient expression^6,19,20^. However, specific modifications to the mRNA nucleotide improve the stability and translational efficiency, prolonging its in vivo half-life, essential for effectiveness as a vaccine^15,21^. mRNA is produced in a cell-free in vitro transcription reaction from a linear DNA template, using RNA polymerase as a catalyst and nucleoside triphosphates (NTPs) as co-substrates^6^, apart from other reaction components^22,23^. Typical production titres are between 2 and 5 g L^−1^^24–27^ but recent studies have shown that titres can be increased to 12 g L^−1^ in batch or fed-batch mode^28,29^.

T7 RNA polymerase (T7 RNAP) has been predominantly used for IVT reactions^30,31^, due to the high fidelity displayed, and consists of a single subunit and is highly processive. The transcription process can however generate double-stranded RNA (dsRNA) contaminants through different mechanisms (e.g. random priming of abortive transcripts^32^, antisense transcription^33^, turn-around transcription and self-primed extension of product RNA^34–36^) which hinder mRNA translation efficiency^37^ and can ultimately compromise vaccine safety^33,38,39^. Therefore, T7 RNAP efficiency and activity are continuously optimised, such as the development of mutant versions that reduce abortive products and immunostimulatory byproducts^40,41^. Additionally, modifying the T7 promoter region facilitates an optimal interaction between T7 RNAP and the DNA template, thereby facilitating the initiation and elongation of transcription, resulting in increased mRNA production^42,43^. Extensive research has been conducted to assess the impact of various T7 promoter variants aimed at increasing total mRNA produced, such as performing modifications in different T7 promoter regions—from the core region^44^ to upstream^45^ and downstream^42^ regions. Nevertheless, these approaches did not explore the T7 RNAP promotor optimisation focusing on the production of mRNA vaccines in a transcription system.

In this contribution, we explored the effect of modifying DNA templates for the synthesis of mRNA in terms of process yield and quality. Site-directed mutagenesis was used in the T7 promoter region with the transcription performance markedly enhanced. The AT-rich insertion in the downstream region of the T7 promoter allowed for a notable increase in mRNA titres compared to the wildtype T7 promoter, reaching a maximum of 14 g L^−1^ in approximately 2 h. The mRNA titres up to 12 g L^−1^ were also achieved in 45 min of IVT reaction, thereby reducing the required reaction time. mRNA quality was increased by minimising the dsRNA concentration as an undesirable byproduct. The results obtained outperformed the wildtype T7 promoter by decreasing the dsRNA production by up to 30%. A decrease was also observed with the increase in template size, but less significant (up to 20%). The modifications the promoter sequence did not alter significantly the initiation of the translation within the cells. The results highlighted the potential of an AT-rich sequence in the downstream region of the T7 promoter as a strategic modification to improve the quantity and quality of mRNA production via in vitro transcription, increasing the cost-effectiveness of mRNA manufacturing^14,23^.

## Material and methods

Unless otherwise stated, all chemicals and reagents were purchased from ThermoFisher Scientific (UK).

### Template construction for mRNA synthesis

#### Template design

The mRNA template consists of the EGFP gene (GenBank Accession #AAB02572.1) flanked by two untranslated regions (5’-UTR and 3’-UTR) and followed by a poly-A sequence. The 5’-UTR comprises three elements: the wildtype or mutant promoter of T7 RNA polymerase, a binding site of eukaryotic translation initiation factor eIF4G, and a Kozak consensus sequence^16,23^. The 3’-UTR utilises two tandem repeats of 3’-UTR from the human β-globin gene. The poly-A sequence (120 bp) is segmented with a 6 bp spacer^46^. Additional templates were assembled by fusing the EGFP gene with the *Klebsiella pneumoniae* transaminase gene (GenBank Accession #AF074934.1), and the EGFP gene with the T7 RNA polymerase gene (GenBank Accession #NP_041960.1). Sequences used in this study are presented in Supporting Information Table S1. All the mRNA templates are inserted in a pUC57 plasmid vector with kanamycin resistance.

#### Promoter modification and plasmid construction

##### Site-directed mutagenesis

Sited-directed mutagenesis was performed to mutate or add downstream and upstream insertion in the promoter region using Q5^®^ Site-Directed Mutagenesis Kit (New England Biolabs, UK). A plasmid control, comprising of mRNA template with wildtype T7 promoter adapted from Rosa et al.^23^ was used as the template for mutagenesis. The amplification of reaction mix (1 × Q5^®^ Hot Start High-Fidelity Master Mix, 0.5 μM forward primer, 0.5 μM reverse primer, and 25 ng plasmid template, and Gibco™ Water for Injection, WFI) was performed through touchdown polymerase chain reaction (TD-PCR) using Applied Biosystems™ Veriti™ 96-Well Thermal Cycler (ThermoFisher Scientific, UK) with the following detailed cycle conditions: an initial denaturation step at 98 °C for 30 s, 10 cycles of 10 s at 98 °C and annealing at 66–57 °C (for samples: T7#4, T7c62, T7c62_T7#4, and T7DI_1 to T7DI_11) or 70–61 °C (for samples: T7Max and T7Max_T7#4) for 30 s. The annealing temperature decreased 1 °C per cycle and an extension step was performed at 72 °C for 30 s per kb. This was followed by 20 cycles of 10 s at 98 °C; annealing at 57.5 °C (for samples: T7#4, T7c62, T7c62_T7#4, and T7DI_1 to T7DI_11) or 61.5 °C (for samples: T7Max and T7Max_T7#4) for 30 s; and extension at 72 °C for 30 s per kb. The final extension was executed at 72 °C for 2 min. Afterwards, 1 μL of TD-PCR products were treated with 1 × Kinase, Ligase, and DpnI (KLD) enzyme mix and buffer (New England Biolabs, UK), and adjusted with WFI to a final volume of 10 μL. The KLD mix was incubated for 5 min at 21 °C e and 5 μL of the mix was used for transformation using a heat shock method. All plasmids and primers used in this study are presented in Supporting Information Table S2 and S3, respectively.

##### Gibson assembly

Using the Gibson assembly method, two plasmids, pT7wt_TA_EGFP and pT7wt_T7 RNAP_EGFP, were obtained. This was achieved by separately integrating the genes for *K. pneumoniae* transaminase (TA) and T7 RNA polymerase (T7 RNAP) into the pT7wt_EGFP template. TA and T7 RNAP genes were amplified and isolated from pET29A_TA and pET29A_T7 RNAP using PCR with specific primers. The linearisation of vector pT7wt_EGFP also performed through PCR. All the PCR reactions were performed using high-fidelity VeriFi™ DNA Polymerase, VeriFi™ Buffer, and VeriMax Enhancer (PCR Biosystems, UK). The PCR products of linear vector pT7wt_EGFP, and isolated TA and T7 RNAP genes were analysed using agarose-gel electrophoresis and the gel containing correct sizes of DNA bands were further isolated and purified. The purified linear vector pT7wt_EGFP, and the insert (TA gene or T7 RNAP gene) were assembled using Gibson Assembly Master Mix (New England Bioscience, UK). For each reaction, the same mass (0.1 pmol) of insert and vector were mixed with 10 μL of 2 × Gibson Assembly Master Mix and adjusted with WFI to a total volume of 20 μL. The reactions were performed at 50 °C for 2 h. Following the incubation, 2 μL of the assembly products were subsequently transformed to *E. coli* NEB 10-beta (New England Biolabs, UK).

#### Molecular cloning

Chemically competent *E. coli* NEB 10-beta cells (New England Biolabs, UK) were prepared by the calcium chloride method and used for routine transformation. Transformation of plasmids was performed using the heat-shock method. Transformed cells were plated on Luria–Bertani agar media (25 g/L Miller LB broth (Sigma-Aldrich) with 15 g/L culture media agar (MP Biomedicals, USA)) with kanamycin (50 µg/mL) and overnight incubated at 37 °C. Colony PCR was performed using high-fidelity VeriFi™ DNA Polymerase with VeriFi™ Buffer and VeriMax Enhancer (PCR Biosystems, UK). Plasmid DNA was purified using GeneJET Plasmid Miniprep Kit, following the protocol by the manufacturer.

#### Plasmid verification and sequencing

NanoDrop™ One Microvolume UV–Vis Spectrophotometer (ThermoFisher Scientific, UK) was used to measure the concentration of purified plasmids. Purified plasmids were digested using EcoRI (New England Biolabs, UK) and LguI/SapI with CutSmart^®^ buffer (New England Biolabs, UK) for one hour incubation at 37 °C, followed by inactivation at 65 °C for 20 min. Approximately 100 ng/µL of the purified plasmids were Sanger sequenced (Eurofins Genomics, UK).

#### Agarose gel electrophoresis

To analyse the PCR and digestion products, 1% (w/v) of agarose (Sigma-Aldrich, USA) was prepared using 0.5 × TBE buffer (45 mM Tris–borate and 1 mM EDTA), Invitrogen SYBR^®^ Safe DNA Gel Stain (1:10,000 dilution), and run at 100 V for one hour. Purple Gel Loading Dye (New England Biolabs, UK) was used to load the samples into the gel and 1 kb Plus DNA Ladder (New England Biolabs, UK) for analysis.

### mRNA and dsRNA synthesis

#### Template production

DNA templates for IVT reactions were produced through touchdown polymerase chain reaction (TD-PCR). The TD-PCR reaction mixture contained between 200 and 250 ng mL^−1^ of plasmid, 0.4 μM of forward and reverse primers, 1 × VeriFi™ Buffer, 1 × VeriMax Enhancer, and 0.02 U μL^−1^ high-fidelity VeriFi™ DNA Polymerase (PCR Biosystems, UK). The reaction mixture was prepared to a total volume of 500 μL and split into 50 μL reaction per tube. Detail of plasmids (as templates) and primers are found in S2 and S3, respectively. Supportive Information Table S. The TD-PCR was performed using a Applied Biosystems™ Veriti™ 96-Well Thermal Cycler (ThermoFisher Scientific, UK) with an initial denaturation step at 95 °C for 1 min, 10 cycles of 15 s at 95 °C; annealing at 60–51 °C (for samples: T7#4 and T7DI_1 to T7DI_11) or 66–57 °C (for samples: T7wt, T7Max, T7c62, T7Max_T7#4, and T7c62_T7#4) for 30 s with annealing temperature decreased 1 °C per cycle. Extension was performed at 72 °C for 30 s per kb, followed by 20 cycles of 15 s at 95 °C; annealing at 51.5 °C (for samples: T7#4 and T7DI_1 to T7DI_11) or 58 °C (for samples: T7wt, T7Max, T7c62, T7Max_T7#4, and T7c62_T7#4) for 30 s and extension at 72 °C for 30 s per kb. The final extension was executed at 72 °C for 2 min. The TD-PCR product was purified using GeneJET PCR Purification Kit (ThermoFisher Scientific, UK) following the manufacturer's instructions. A 10 × concentrated TD-PCR product was obtained from the purification step and further quantified using NanoDrop™ One Microvolume UV–Vis Spectrophotometer (ThermoFisher Scientific, UK).

#### In-vitro transcription (IVT) reactions

The IVT reaction conditions were adapted from Rosa et al. (2022). The IVT reaction mixture contained 89 nM of linear DNA template (purified TD-PCR product), 7.75 mM of each NTP (ATP, GTP, CTP, and UTP), 5.3 mM of DTT, 49 mM of Mg-acetate (Santa Cruz Biotechnology, USA), 40 mM pH 6.5 Tris buffer, 2.3 mM of spermidine, 0.008 U μL^−1^ *of Saccharomyces cerevisiae* inorganic pyrophosphatase, 1.48 U μL^−1^ of RiboShield™ RNase Inhibitor (PCR Biosystems, UK), 7.7 U μL^−1^ of bacteriophage T7 RNA polymerase, and was made up to a final volume of 20 μL (for sample measurements) or 100 μL (for the calibration curve) with water for injection (WFI). The IVT was performed at 43 °C for 2 h. The mRNA produced from IVT was quantified using reverse‐phase high‐performance liquid chromatography (RP‐HPLC) described in Section “mRNA quantification”.

To produce dsRNA for the calibration curve, a subsequent incubation was performed after IVT to facilitate the dsRNA hybridisation. The 100 μL of dsRNA IVT product was diluted to 200 μL with water for Injection (WFI) and incubated at gradient temperature 85 °C to 23 °C for 2 min at each temperature.

#### RNA purification

The mRNA purification for the calibration curve was performed using MEGAclear™ Transcription Clean-Up Kit as instructed by the manufacturer with slight modifications. The 100 μL of IVT product was treated with 2 μL of TURBO™ DNase and incubated for 15 min at 37 °C. For dsRNA purification, after the hybridisation incubation step, 2 μL of TURBO™ DNase and 2 uL of RNase T1 were added to 200 μL of diluted dsRNA IVT product and incubated at 37 °C for 15 min. The 350 μL of binding solution and 250 μL of 100% v/v ethanol were added to the samples and then loaded into the filter cartridge for centrifugation (15,000 × g, 1 min, 21 °C). The filter was washed with 500 μL of wash solution and centrifuged under the same condition in the previous step twice. The mRNA was eluted with 50 μL of elution buffer, followed by 5 min incubation at 65 °C and centrifugation at 15,000 × g for 1 min at 21 °C. The elution step was repeated in the same previous condition. The 100 μL of purified mRNA was further precipitated with 10 μL of 5 M ammonium acetate and 275 μL of 100% v/v ethanol, and then overnight incubated at − 20 °C. Samples were centrifuged (top speed) at 4 °C for 15 min. The supernatant was discarded, and the obtained pellet was air-dried to remove the remaining ethanol. The pellet was resuspended in 40 μL of elution buffer. The concentrated purified mRNA sample was then quantified using NanoDrop™ One Microvolume UV–Vis Spectrophotometer (ThermoFisher Scientific, UK) and RP-HPLC (Section “mRNA quantification”).

#### mRNA capping for expression studies

One-pot cap-1 reactions were performed using Faustovirus Capping Enzyme (New England Bioloabs, UK) and Cap 2’-O-methyltransferase (New England Bioloabs, UK). Briefly, 50 µg of purified mRNA was added to a reaction containing 1X FCE capping buffer, 0.5 mM GTP, 2 mM S-adenosylmethionine, 1 µL of Rnase inhibitor, 1 U µL^−1^ of Faustovirus Capping Enzyme, 4 U µL^−1^ of Cap 2’-O-methyltransferase, and WFI water to a final volume of 50 µL. The samples were incubated at 37ºC without shaking for 2 h. Afterwards, the samples were purified as described in Section “rna purification”. The pellets were resuspended in 10 µL of WFI water, and quantified using NanoDrop™ One Microvolume UV–Vis Spectrophotometer (ThermoFisher Scientific, UK).

### Analytical methods

#### mRNA quantification

##### RP-HPLC

The total mRNA concentration was quantified using the established RP‐HPLC gradient method adapted from Issa and Packer^47^ and Rosa et al.^23^. An UltiMate™ 3000 UHPLC System with a 2.1 × 100 mm DNAPac™ RP column and a 3 × 10 mm guard column was used. 5 µL of each sample, diluted 6 times, was run in the pre‐equilibrated column with TAE buffer absorbance measured at 260 nm. Elution was achieved by a gradient elution using TAE buffer with 25% acetonitrile. The runs were performed at 80 °C with the following conditions: After injection, the column is washed for 1 min and 0.2 mL × min^−1^. The flow is gradually increased to 0.25 mL × min^−1^ for 30 s. A gradient to 6% of elution buffer and 0.35 mL × min^−1^ is applied for 30 s, followed by a gradient to 76.5% of elution buffer at 0.4 mL × min^−1^ for 4 min, and a final gradient to 100% elution buffer for 1 min. The column is then washed with 100% elution buffer for 3 min, and re-equilibrated with the binding buffer for 6 min.

##### Agarose gel electrophoresis

A 2% (w/v) of agarose with 0.5 × TBE buffer (45 mM Tris–borate and 1 mM EDTA) and 5.5 mM of magnesium chloride was prepared and pre-stained with Invitrogen SYBR^®^ Safe DNA Gel Stain (1:10,000 dilution). The gel was loaded with 1.5 μL of mRNA sample diluted in WFI into a final volume of 10 μL and 2 μL of 6 × purple Loading Dye (New England Biolabs, UK). A 5 μL of 1 kb Plus DNA Ladder (New England Biolabs, UK) was used as the molecular marker. The electrophoresis was run at 100 V for 75 min using 0.5 × TBE buffer containing 5.5 mM of MgCl_2_. The gel was visualised using Amersham™ Imager 600 (GE Healthcare, UK).

#### dsRNA quantification

The dsRNA concentration was measured using the RP‐HPLC method adapted from Issa et al.^47^ described in Section “mRNA quantification”. Samples of 10 μL were treated with 0.5 μL of RNase T1 and incubated at 37 °C for 15 min to digest the ssRNA (Supplementary Information Fig. S2).

### Protein expression

EGFP expression was performed using the 1-Step Human Coupled IVT kit. Capped mRNA (Section “mRNA capping for expression studies”) was diluted to a final concentration of 1 g L^−1^ and 2 µL were added to the reaction mixture. Positive and negative controls were the kit GFP control and WFI water, respectively. The samples were incubated for 6 h at 30 °C without agitation. The samples were diluted 1:2 with WFI water to a final volume of 50 µL and the EGFP fluorescence was measured using Infinite Pro 200 (Tecan, USA).

### Statistical analysis

Statistical analyses were conducted using GraphPad Prism (version 10.0.2): one-way ANOVA with the Brown-Forsythe test (determine the standard deviation for duplicate IVT experiments for mRNA concentration and dsRNA level across promoter variants), Dunnett’s and Tukey’s multiple comparisons test (compare the total mRNA concentration and the dsRNA level across promoter variants) and two-way ANOVA followed by Tukey’s multiple comparisons test (analysing and comparing the mRNA concentration and dsRNA level in T7 promoter variants over a range of template sizes).

## Results

### T7 promoter modifications

In this study, we evaluated a total of 16 different T7 promoter variants, detailed in Table 1. For comparison, three reported T7 promoter variants were used as a positive control, namely T7#4^42^, T7Max^43^, and T7c62^44^, were selected based on their improved transcriptional performance relative to the wild-type T7 promoter both in IVT systems and cell-free transcription/translation systems. Each control exhibits a modification in a specific region of the T7 promoter, namely in the upstream, downstream, or within the core promoter region. Particularly, the T7c62 variant carries nucleotide substitutions within the core promoter region, at positions -4 (A substituted for T), − 1 (C for A), and + 2 (A for G). This variant was reported to demonstrate approximately twofold higher protein expression level than wildtype T7 promoter^44^ in cells. The specific sequences in both upstream (− 22 to − 18)^43,45^ and downstream (+ 4 to + 8)^42^ regions of the T7 promoter also have been documented to improve the transcription levels. T7Max incorporates an upstream element (AATTC) at positions − 22 to − 18, which has been linked to increased gene expression in in vitro systems^43^. T7#4 contains an AT-rich downstream element, ‘ATAAT’, at positions + 4 to + 8^42^. This promoter was employed as a representative variant containing an AT-rich downstream element, previously demonstrated to improve T7 promoter activity, with amplicon abundances increasing by over a fivefold range compared to GC-rich combinations^42^. T7Max_T7#4 and T7c62_T7#4 promoters were also constructed to evaluate the synergistic effects of incorporating modifications from disparate regions. Furthermore, a library of AT-rich downstream variants that are composed by 11 variants, T7DI_1 to T7DI_11, and that contains different AT-rich combinations at positions + 4 to + 8, was created. The variants T7DI_1 to T7DI_11 aimed to assess the effects of alternate AT-rich sequences and evaluate the sequence-specificity of these elements on modulating transcriptional activity. It is hypothesised that AT-rich sequences can facilitate DNA unwinding during the initiation of the transcription.

**Table 1:**
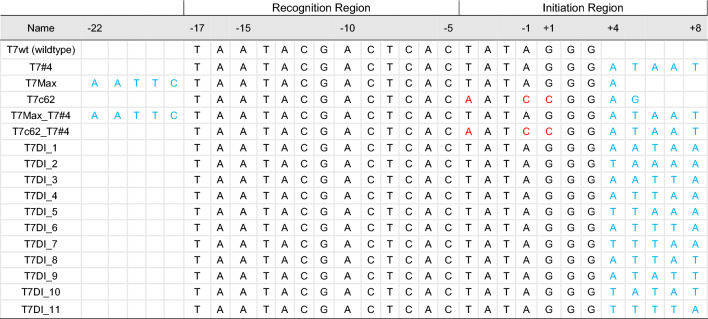
T7 promoter variants used in this study.

The sample ID for the T7 promoter variants T7#4^42^, T7Max^43^, and T7c62^44^ references the original. T7Max_T7#4 and T7c62_T7#4 combine both T7#4 sequence with the T7Max and T7c62 modifications. 11 promoter variants in this study are designated with the prefix “T7DI”, followed by a numerical identifier. T7DI corresponds to T7 promoter with a specific downstream sequence, while the accompanying number indicates the unique arrangement of AT-rich sequences located at positions + 4 to + 8 in the downstream region. In blue: added sequences, and in red: substituted sequences.

### Impact of T7 promotor modification on IVT performance

#### Specific AT-rich downstream elements

**Figure 1:**
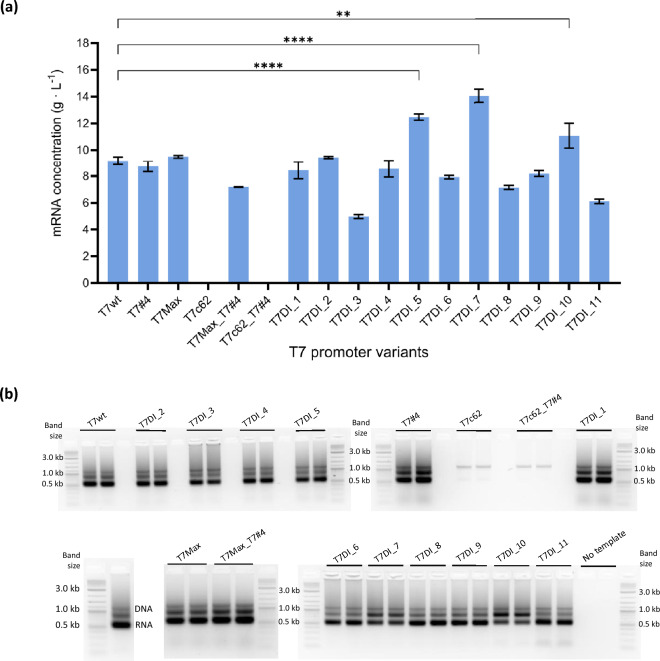
The mRNA production profiles of the different T7 promoter variants. IVT reactions were performed according to Rosa et al.^23^ at 43 °C for 2 h. (**a**) Total mRNA produced and quantified by RP-HPLC. Error bars represent the standard deviation for n = 2. One-way ANOVA with Brown-Forsythe test and followed by Dunnett’s multiple comparisons test, ** and **** denote *p*-values of 0.0012 and < 0.0001 respectively. (**b**) Agarose-gel electrophoresis analysis of IVT products. The mRNA produced is indicated by the 600 nt RNA band while the 1.2 kb band represents the linear DNA template used. Gels do not present normalised mRNA quantity, and the variances observed are a result of independent IVT reactions. The cropped gel images are displayed to improve clarity and conciseness. The original gels are available in Supporting Information Fig. S1.

The linear DNA templates encoding for EGFP with the T7 promoter variant were used for in vitro transcription (Fig. 1). Several promoter variants from the library designed that contain specific AT-rich downstream sequences significantly outperformed the wild-type T7 promoter (T7wt), producing at least 10 g L^−1^ within 2 h (Fig. 1a). T7DI_7 achieved the highest mRNA concentration at 14.05 ± 0.5 g L^−1^, marking approximately 1.5-fold increment relative to T7wt (9.18 ± 0.29 g L^−1^). This was followed by T7DI_5 and T7DI_10 with 1.4-fold and 1.2-fold mRNA yield compared to T7wt. Several promoter variants, i.e. T7DI_1, T7DI_2, T7DI_4, and T7DI_9, produced similar amounts of mRNA as T7wt, ranging from 8.24 to 9.45 g L^−1^ (*P* > 0.05). In contrast, the other variants exhibited lower mRNA yield compared to T7wt, with T7DI_3 showing the lowest concentration at 4.97 ± 0.15 g L^−1^ (*P* < 0.05) (Fig. 1a).

The control T7 promoter variants T7#4^42^ and T7Max^43^ exhibited no significant differences in mRNA production compared to T7wt (*P* > 0.05,). However, the T7Max_T7#4 promoter, which combines modifications from both upstream (-22 to -18) and downstream (+ 4 to + 8) regions produced lower mRNA levels (7.19 ± 0.02 g L^−1^) indicating the possible counteractive effects of combined elements (Fig. 1a). The T7c62^44^ and T7c62_T7#4 promoters produced no mRNA (Fig. 1a,b). Although T7c62 was previously reported to demonstrate higher expression level^44^, no mRNA was produced after 2 h of IVT in this study. A similar result was also reported with no activity for the RNA broccoli aptamer transcribed using the T7c62 promoter variant^43^.

#### Kinetic analysis of T7 promoter modification and impact on production yields

**Figure 2:**
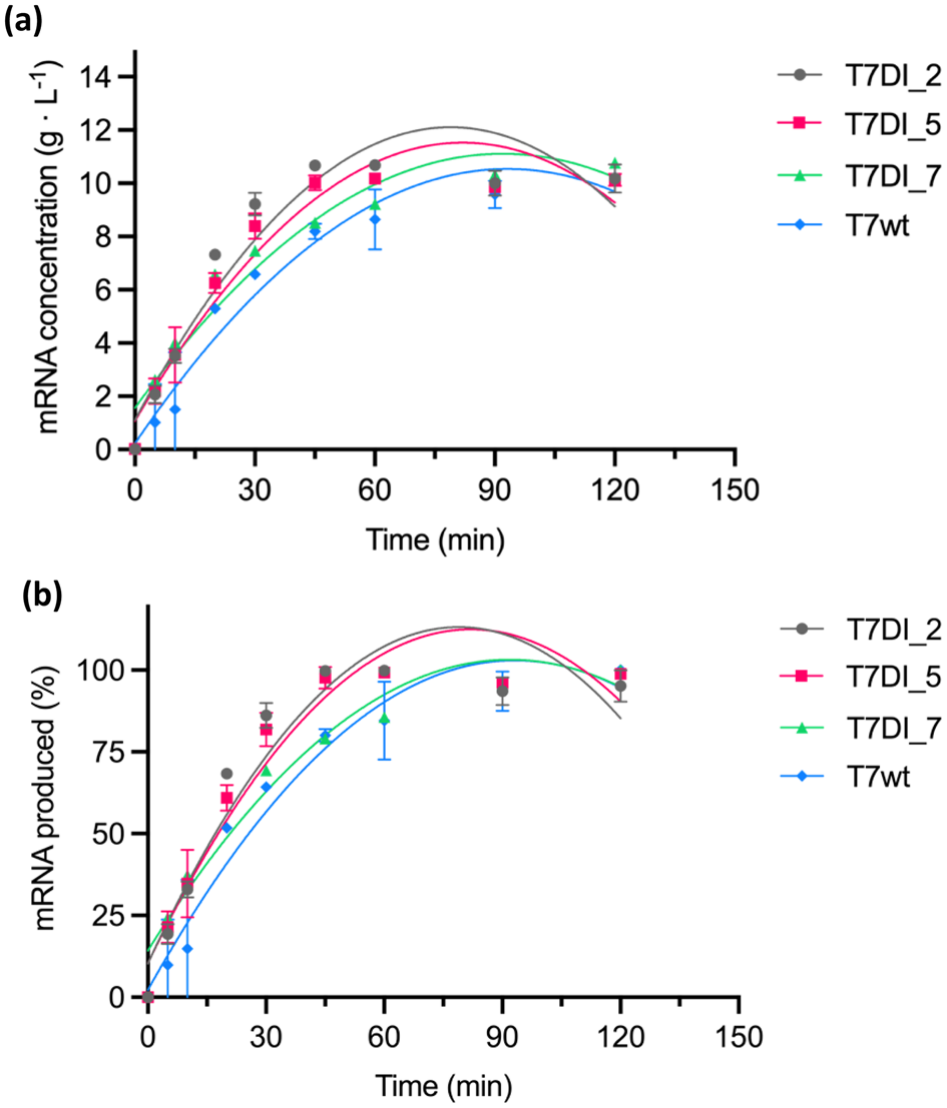
The kinetics analysis of T7 promoter variants. IVT reactions were performed according to Rosa et al.^23^ 43 °C for 2 h. (**a**) mRNA concentration (g L^−1^) for a time course of 2 h. (**b**) mRNA produced as a function of reaction time, with the maximum mRNA concentration achieved corresponding to 100%. Error bars represent the standard deviation for n = 2 replicates. The lines correspond to a second order polynomial (quadratic) function.

Three promoter variants were selected for subsequent kinetic analyses based on the production profile during the screening phase, namely final concentration of approximately 10 g L^−1^ after 2 h (Fig. 1). The T7DI_5, and T7DI_7 promoters achieved higher production yields compared to T7wt, and T7DI_2 presented the least variance in production yield. The reaction profile shows that T7DI_2 and T7DI_5 reached a sheiling of total mRNA produced in 45 min, achieving maximum concentrations of 10.67 ± 0.06 and 10.01 ± 0.28 g L^−1^, respectively. After 120-min reaction time, no significant differences in total mRNA concentrations were observed for T7DI_2 and T7DI_5 promoters (Fig. 2). In contrast, T7DI_7 exhibited a lower production rate than T7DI_2 and T7DI_5, but still outperforming T7wt. Initial kinetic measurements within the first 15 min indicated similar production rates among T7DI_2, T7DI_5, and T7DI_7. After the 15-min time point, T7DI_7 showed a diminished rate relative to T7DI_2 and T7DI_5, followed by a gradual increase in mRNA production, peaking at the 120-min mark. In contrast, no such increase in mRNA yield was observed for T7DI_2 and T7DI_5 post-45 min. The findings demonstrate that, following a 2 h incubation in IVT, the promoter variants produced comparable quantities of mRNA regardless of the different transcriptional rates. This time frame is in line observations obtained with optimised IVT conditions^23,48^.

#### T7 promoter variants with specific AT-rich downstream elements produce less dsRNA byproduct

The evaluation of T7 promoter candidates focused on achieving higher mRNA yields whilst reducing dsRNA impurities (Table 2).

**Table 2:** The comparison of total mRNA and dsRNA concentrations for three T7 promoter candidates.

Promoter candidates	Total mRNA (g L^−1^)	dsRNA/mRNA total (mg g^−1^)
T7wt (wildtype)	9.18 ± 0.29	181.34 ± 1.32
T7DI_2	9.45 ± 0.08	96.51 ± 1.46
T7DI_5	12.47 ± 0.23	138.47 ± 1.08
T7DI_7	14.05 ± 0.50	92.22 ± 2.84

The IVT reactions were prepared following methods previously described^23^ and the reactions were conducted at 43 °C for 2 h.

**Figure 3:**
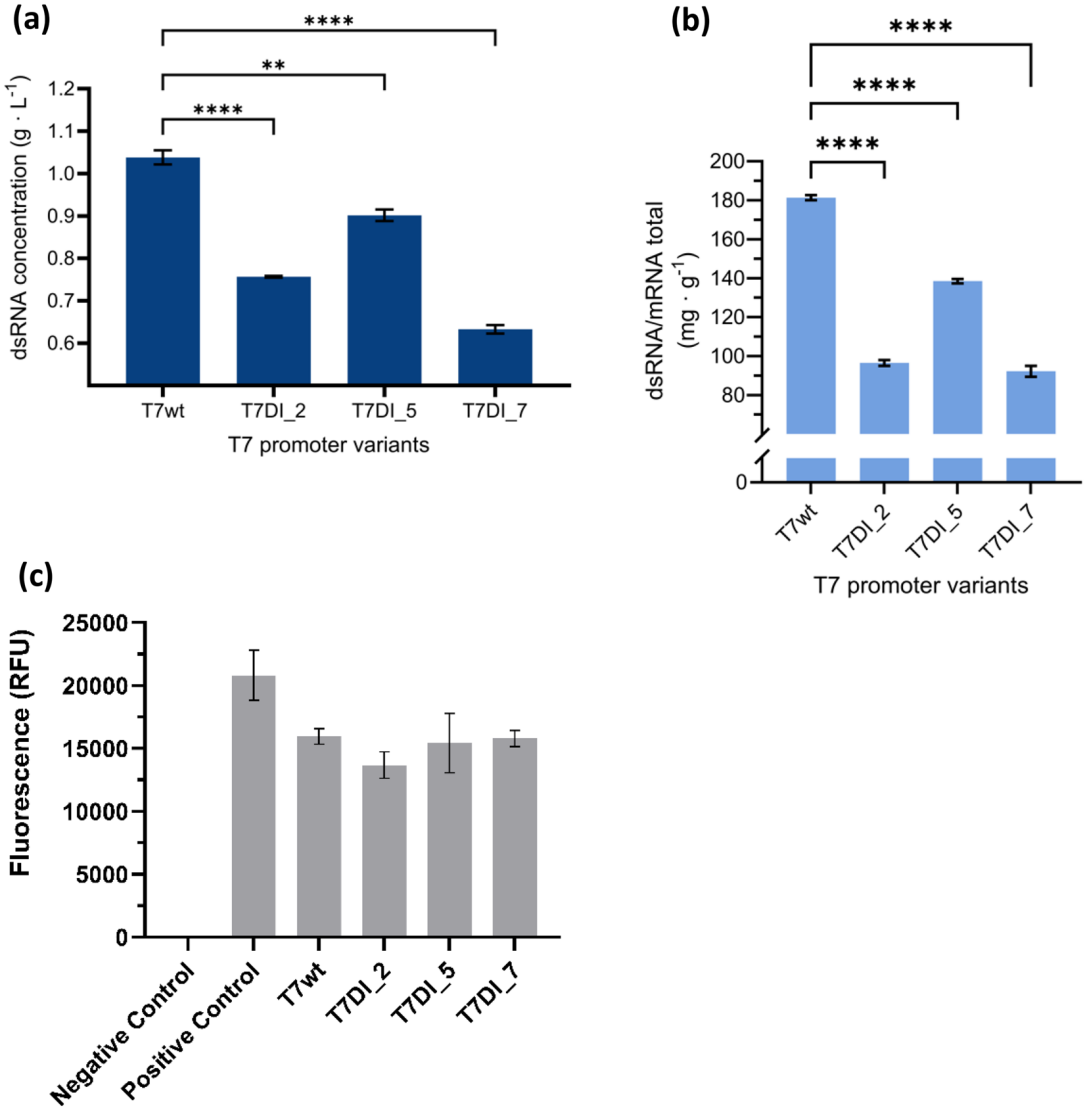
dsRNA production and EGFP protein expression of the different T7 promoter variants. The IVT reaction mix was prepared based on Rosa et al.^23^ and the reactions were performed at 43 °C for 2 h. Prior to quantification, RNase T1 was added to each IVT product to degrade the ssRNA. (**a**) dsRNA concentration (g L^−1^) quantified by RP-HPLC. (**b**) Concentration of dsRNA per gram mRNA (mg g^−1^) produced during IVT. Error bars represent the standard deviation (n = 2). (**c**) EGFP expression (RFU). Positive and negative controls are the kit GFP control and WFI water, respectively. One-way ANOVA with Brown-Forsythe test followed by Tukey’s multiple comparisons test, ** and **** denote *p*-values of 0.0012 and < 0.0001, respectively. No significant differences (*p* > 0.05) do not have *p*-value annotations.

Prior to quantification, the IVT products were treated with RNase T1 to degrade the single-strand RNA (ssRNA). The T7 promoter variants T7DI_2, T7DI_5, and T7DI_7, which contain specific AT-rich downstream elements, produced significantly lower amounts of dsRNA byproduct compared to T7wt. Among these, T7DI_7 showed the minimal residual dsRNA at a concentration of 0.63 ± 0.07 g L^−1^, a 39.4% reduction in comparison to T7wt (1.04 ± 0.08 g L^−1^). T7DI_2 and T7DI_5 followed with dsRNA concentrations of 0.76 ± 0.06 g L^−1^ and 0.9 ± 0.07 g L^−1^, marking a reduction of 27.1% and 13.2% respectively, relative to T7wt. T7DI_2, T7DI_5, and T7DI_7 also exhibited lower ratios of dsRNA byproduct per gram of total mRNA produced compared to T7wt. T7wt produced dsRNA at a ratio of 181.34 ± 1.32 mg g^−1^, corresponding to approximately 18% of the total mRNA produced (Fig. 3b). T7DI_5 showed a decrease of 23.6% in the dsRNA/mRNA_total_ ratio compared to T7wt, with a ratio of 138.47 ± 1.08 mg g^−1^ (13.85% of the total mRNA produced). In addition, both T7DI_2 and T7DI_7 achieved a 46–49% reduction in dsRNA concentration compared to T7wt and were approximately 30–33% lower than T7DI_5 (Fig. 3b). Modifications of the promoter sequence were evaluated also by EGFP protein expression (Fig. 3C). No significant differences were observed, indicating that the modifications downstream to the promoter have no significant effect on the initiation of the translation within the cells.

### Evaluation of different sizes of templates and the impact on IVT

**Figure 4:**
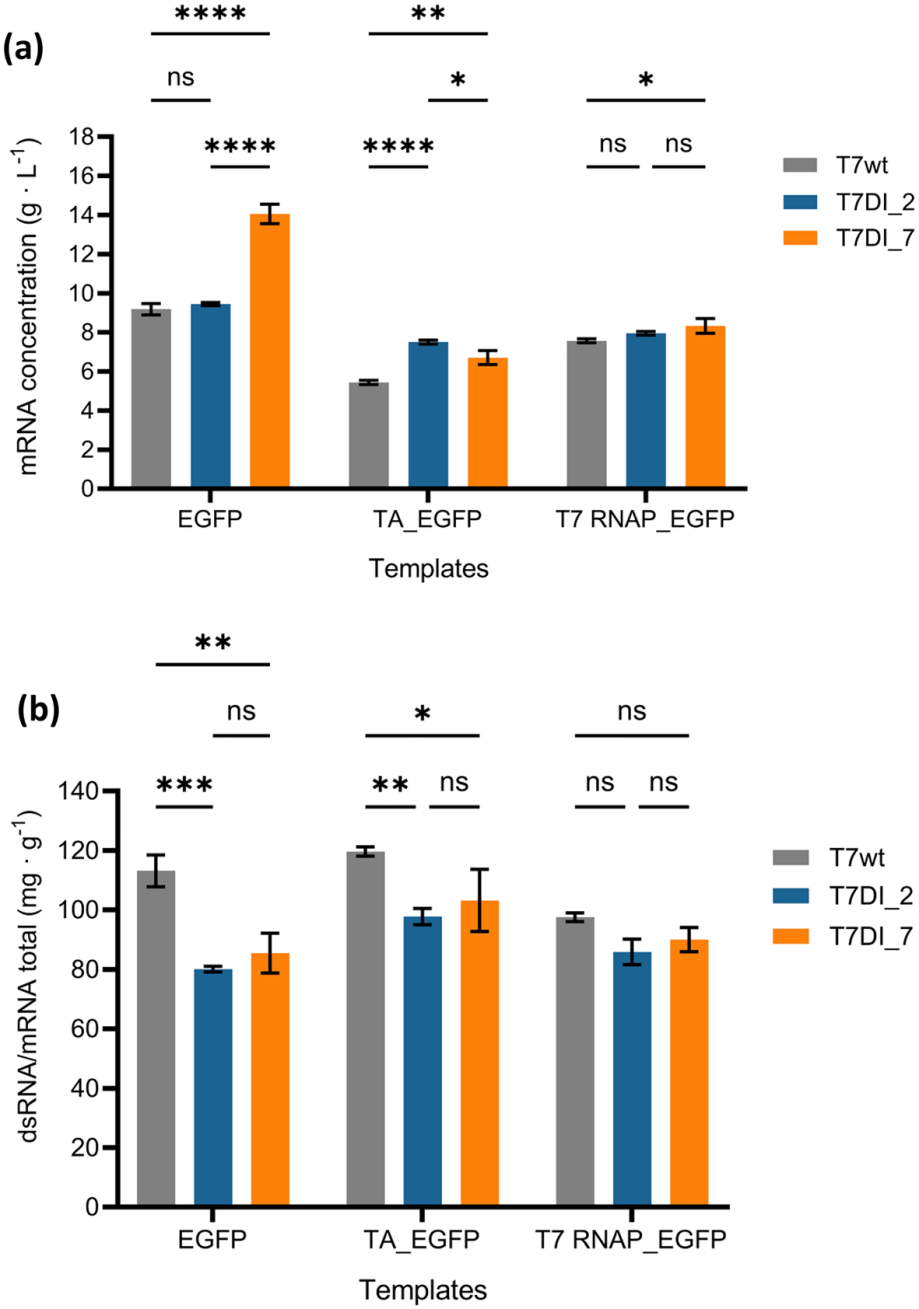
The profiles of mRNA production and dsRNA byproduct in T7 promoter variants across diverse template sizes. The IVT reaction mix was prepared based on Rosa et al.^23^. Two hours of IVT reactions were performed using T7 promoter variants (T7wt, T7DI_2, and T7DI_7) with three different sizes of templates (EGFP—1195 bp; TA_EGFP—2483 bp; T7 RNAP_EGFP—3851 bp). The mRNA and dsRNA concentrations were quantified using RP-HPLC. (**a**) The mRNA production (g L^−1^) and (**b**) the ratio of dsRNA byproduct per gram mRNA total (mg g^−1^) across different sizes of templates. For a–b: error bars represent the standard deviation for n = 2 replicates. Two-way ANOVA followed by Tukey’s multiple comparisons, the significance levels are indicated as follows: ‘ns’ means no significant difference (*p* > 0.05), * for *p*-values between 0.01 and 0.045, ** for *p*-values between 0.001 and 0.005, *** for *p*-value of 0.0003, and **** for *p*-values of < 0.0001. IVT, in vitro transcription; EGFP, enhanced green fluorescence protein; TA, Transaminase; T7 RNAP, T7 RNA polymerase; RP-HPLC, reverse phase-high performance liquid chromatography.

The mRNA production profiles of T7 promoter variants were evaluated using three different sizes of templates: 1195 pb (containing EGFP gene), 2483 bp (fused *Klebsiella pneumoniae* transaminase and EGFP genes) and 3851 bp (fused T7 RNA polymerase and EGFP genes) (Fig. 4a). The corresponding promoter sequences that produced the lowest amount of dsRNA were chosen. For the 1.2 kb EGFP template, the T7DI_7 promoter produced 14.05 ± 0.5 g L^−1^, representing a 1.5-fold increase in comparison to T7wt, which produced 9.18 ± 0.29 g L^−1^. In contrast, the T7DI_2 produced 9.45 ± 0.08 g L^−1^, showing no statistically significant difference from the T7wt (*P* > 0.05). However, with the larger 2.5 kb TA_EGFP template, T7DI_2 produced 7.50 ± 0.10 g L^−1^, outperforming both T7DI_7 and T7wt by 1.12-fold and 1.38-fold, respectively (*P* < 0.05). For the 3.9 kb T7 RNAP_EGFP template, no significant differences in mRNA production were observed between T7wt and T7DI_2, or between T7DI_2 and T7DI_7 (*P* > 0.05). Changing the template size from 1.2 kb to 2.5 kb influenced the mRNA yields across T7 promoter variants. For the 2.5 kb TA_EGFP template, T7DI_7 exhibited a 52.3% decrease, followed by 40.7% and 20.6% reductions in T7wt and T7DI_2, respectively, when compared to the 1.2 kb EGFP template (*P* < 0.001). A similar trend was also observed when comparing the 3.9 kb (T7 RNAP_EGFP) template to the 1.2 kb (EGFP) template, marking decreases in mRNA production in T7 RNAP_EGFP by 40.7%, 17.6%, and 15.8% for T7DI_7, T7wt, and T7DI_2, respectively (*P* < 0.001). Rosa et al. (2022) also reported that a larger 5.3 kb template, encoding fused Cas9 and EGFP genes, performed a 27% reduction in final mRNA concentration after a 2 h reaction compared to a smaller 1.2 kb template encoding EGFP gene^23^. However, changing the template size from 2.5 kb (TA_EGFP) to 3.9 kb (T7 RNAP_EGFP) did not significantly impact the mRNA production. T7DI_2 consistently produced 7.5 to 7.9 g L^−1^, with modest increases observed in T7wt and T7DI_7. Among the T7 promoter variants examined, T7DI_2 exhibited minimal variation in mRNA yield across different templates.

The dsRNA profile was also evaluated for the three different sizes of mRNA. In the 1.2 kb EGFP template, T7wt exhibited the highest dsRNA byproduct level at a ratio of 113.15 ± 5.36 mg per gram of mRNA total (mg g^−1^) (Fig. 4b). These ratios were approximately 1.4-fold and 1.3-fold higher than those produced by T7DI_2 and T7DI_7, respectively. The same trend was observed in larger templates, although with lower degree. Interestingly, the effect decreases with the increase of template size. The dsRNA/mRNA_total_ ratio was reduced by 18% in the 2.5 kb TA_EGFP template compared to the 1.2 kb EGFP template (Fig. 4b). Changing the template size to 3.9 kb (T7 RNAP_EGFP) in T7wt also reduced the dsRNA/mRNA_total_ ratio by 12% relative to EGFP. T7DI_2 also demonstrated to have a stronger impact on dsRNA byproduct levels compared with T7DI_7.

## Discussion

T7 RNA polymerase (RNAP) is the enzyme of choice to be used in the IVT system, owing to its simple structure, efficient production of long transcripts, and high specificity towards the T7 promoter^30,49^. Improving IVT system productivity may rely on improving transcriptional efficiency and mRNA quality. Different strategies can be used to lower dsRNA. Recently, an engineered T7 RNAP has demonstrated a significant reduction in dsRNA production^40^. However, this mutant produces less total RNA compared to the WT. Through promoter optimisation, a 50% reduction was achieved in dsRNA produced, compared to WT, (Fig. 3) while increasing mRNA production yields (Fig. 1) and rates (Fig. 2). Therefore, optimising T7 promoter sequences emerges as a viable and efficient strategy for enhancing transcription performance and minimizing dsRNA impurities during IVT reactions.

In this work, we assessed the effect of modification in the promoter regions in the overall yield of the IVT reaction. We compared 11 variants that contained a AT-rich region at the promoter downstream, and modifications previously reported to enhance T7 RNAP transcriptional performance were used as controls. It is posited that the AT-rich element in the downstream region (+ 4 to + 8) may facilitate DNA template unwinding and initiate the transcription bubble^42^, thus enhancing the transcription performance and total mRNA produced. Extended AT-rich sequences in the upstream region have been shown to enhance the stability of the polymerase-promoter complex by inhibiting dissociation^45^ and improve the in vitro protein synthesis^43^, whereas downstream AT-rich motifs facilitate the unwinding of the DNA double helix during transcription initiation^42^.

Several promoter variants outperformed the wild type 77 promoter, namely T7DI_7 (1.5-fold increment), T7DI_5 (1.4-fold) and T7DI_10 (1.2-fold). Nonetheless, the downstream AT-rich sequences produced varied mRNA concentrations. The higher production rates observed funderline the positive effect of the AT-rich downstream element at positions + 4 to + 8, which may facilitate the unwinding of the double-stranded DNA template and the initiation of a transcription bubble which commences at the − 4 position and extends downstream^42,50^. Lower production yields are also observed. The reduced mRNA yield observed in T7Max_T7#4, underscores the importance of further investigation into the cumulative effects of these multiple promoter modifications. The T7c62 promoter contains mutations at positions − 4 (A for T), − 1 (C for A), and + 2 (A for G)^44^. These positions encompass the TATA sequence from − 4 to − 1, which serves as the unwinding region and plays a pivotal role in the formation of transcription bubble^50^. Nucleotides at positions − 1 (A) and − 4 (T) are highly conserved across bacteriophage promoters and are notably AT-rich^51^. Furthermore, templates with a guanine (G) triplet at positions + 1 to + 3 of the T7 promoter were transcribed more robustly and may prevent premature dissociation of abortive transcripts^42^. These observations suggest that mutations at positions − 4, − 1, and + 2 within T7c62 might have a profound effect on transcriptional activity, potentially explaining the lack of mRNA produced.

While the varied promoter modifications might produce similar mRNA concentrations, their underlying mechanism by which transcription performance is modulated could be different. The different transcriptional rates in T7DI_2, T7DI_5, and T7DI_7 might arise from distinct AT combinations which highlights the sequence-specific manner of the AT-rich downstream element. Nonetheless, the mechanism underlying the distinct effects of AT combinations on transcription rates remains unknown and requires further investigation.

Considering the reduction in the dsRNA levels, it suggests that the AT-rich downstream elements may influence the stability of the transcription initiation complex and minimize the generation of abortive transcripts. During the initiation of transcription, T7 RNAP binds to the T7 promoter and synthesizes short RNAs or abortive transcripts via a mechanism known as abortive cycling^52–54^. These abortive transcripts can either anneal to each other or interact with T7 RNAP through RNA-templated transcriptional capabilities, giving rise to short dsRNA molecules^34,35,53^. Therefore, minimizing the formation of abortive transcripts during transcription initiation potentially influences the concentration of dsRNA byproduct produced in the IVT reaction. AT- rich sequences can have an impact on the stabilisation of the T7 RNAP-DNA complex^45^.AT-rich sequences in the upstream region of the T7 promoter have been reported to increase the stability of the polymerase-promoter complex by reducing the dissociation rate constant^45^. The AT-rich sequences in the downstream region might exert similar stabilizing effects on the initiation complex, which may influence the levels of dsRNA by-product. Additionally, varying dsRNA concentrations among the promoter variants with AT-rich downstream elements also highlight the sequence-specific characteristic of this element.

The variability in final mRNA concentrations across various template sizes suggests that the mRNA production is potentially influenced by the specific gene sequences encoded within the template rather than by the size, highlighting the sequence-dependent factors in transcriptional efficiency. Specific sequence characteristics have been implicated in the formation of abortive transcripts or truncated mRNA species, which can affect the efficiency of full-length mRNA synthesis^52,53^. In addition, the presence of specific sequences that are energetically favourable to RNA dimerization can trigger the formation of self-complementary mRNA^35,53^. In this condition, the mRNA duplex can interact with RNA polymerase through RNA-templated transcription capabilities, thereby influencing the production of canonical mRNA molecules^34,35,55^.

The dsRNA level varies with different lengths of mRNA^40^. The percentage of dsRNA produced per total mRNA (% w/w) is reported to decrease from approximately 7.5% in ~ 850 nt mRNA to 2% in ~ 1500 nt mRNA^40^. However, a modest increase to roughly 4% for ~ 2900 nt mRNA was observed compared to ~ 1500 nt mRNA^40^. The variations in dsRNA levels across different templates suggest that formation of extended loopback dsRNA species^34,35^. Nevertheless, longer templates will produce dsRNA formation is a sequence-dependent mechanism^52,53^. mRNA can also act as templates for T7 RNA polymerase, leading to the lower amounts of mRNA strands. It is also noteworthy that the formation of abortive transcripts is sequence-specific^52,53^. The dsRNA formation is potentially more influenced by particular sequences encoded in the template that encourage the formation of RNA dimers^35,53^ than by the size of the gene itself.

The relatively lower levels of dsRNA exhibited by T7DI_2, along with its consistent performance across templates, suggest that a specific AT-rich downstream element may influence the dsRNA formation through mechanisms related to abortive cycling. The synthesis of short dsRNA species and abortive transcripts is associated with the initiation phase of the transcription^36,52^. Considering that the position of this element is at + 4 to + 8 downstream of the T7 promoter, where the initiation of transcription bubble occurs^42,50^, it might stabilize the initiation-to-elongation transition during transcription, thereby reducing the number of abortive cycles. Nevertheless, this hypothesis needs further investigation.

## Conclusions

We demonstrated that modifications to the DNA template sequence have improved mRNA yields and quality. An AT-rich region at the downstream of the promoter resulted in an increase in the mRNA production of 14 g L^−1^ in approximately 2 h. Analysing the mRNA production profile, it is observed that the promoter variants peak the mRNA production at 45 min, impacting overall production times. The increase in mRNA production is accompanied by a reduction in dsRNA formation of at least 18% mostly due to a reduction in abortive cycling. Similar observations were made when with different pDNA templates (~ 1200 bp to ~ 3900 bp). The optimisation of the non-coding regions sequences can lead to a positive impact on vaccine effectivity and stability but also to increase production yields and product quality during vaccine manufacturing. This is of paramount importance if a rapid response is required in events of future epidemics with quality on-demand productions. An increase in mRNA quality with a reduction in intensive purification operations will undoubtedly influence the manufacturing process’s cost-effectiveness, ultimately making these vaccines affordable to all.

### Supplementary Information

Supplementary Information.

## Data Availability

The T7 promoter sequences tested in this study are available in Table 1. This study utilized reported promoter variants T7#4^42^, T7Max^43^, and T7c62^44^ as shown in Table 1. The sequences used in this study are provided in the Supporting Information Table S1. The EGFP gene, T7 RNA polymerase gene, and *Klebsiella pneumoniae* transaminase gene sequences used in this study are available in the NCBI GenBank accession numbers NP_041960.1, AAB02572.1, and AF074934.1, respectively. All source and generated data are provided in the paper.
