# Supplementary material for: Comprehensive evaluation of T7 promoter for enhanced yield and quality in mRNA production
Source: Sci Rep. 2024 Apr 26;14:9655. doi: 10.1038/s41598-024-59978-5 (PMC11053036; doi:10.1038/s41598-024-59978-5)
Supplement: Supplementary file 1 — Supplementary Information. [file 41598_2024_59978_MOESM1_ESM.docx]

# Supporting Information

**Comprehensive evaluation of T7 Promoter for Enhanced Yield and Quality in mRNA Production**

Yustika Sari ^a #^, Sara Sousa Rosa ^a,b,c #^, Jack Jeffries ^a^, Marco PC Marques ^a *^

^a^ Department of Biochemical Engineering, University College London, Gordon Street, London,

WC1E 6BT, United Kingdom

^b^ Department of Bioengineering, iBB—Institute for Bioengineering and Biosciences, Instituto

Superior Técnico, Universidade de Lisboa, Lisboa, Portugal

^c^ Associate Laboratory i4HB—Institute for Health and Bioeconomy, Instituto Superior Técnico, Universidade de Lisboa, Lisboa, Portugal

# These authors contributed equally to this work

**Table S1.** Sequences of the the 5’-UTR, 3’-UTR, poly-A, and the gene of interest for DNA templates EGFP, TA_EGFP, and T7 RNAP_EGFP, used in this study.

| **Name** | **Size (bp)** | **Sequence** |
| --- | --- | --- |
| 5’-UTR | 45 | ACTCACTATTTGTTTTCGCGCCCAGTTGCAAAAAGTGTCGCCACC |
| 3’-UTR | 284 | GAGAGCTCGCTTTCTTGCTGTCCAATTTCTATTAAAGGTTCCTTTGTTCCCTAA  GTCCAACTACTAAACTGGGGGATATTATGAAGGGCCTTGAGCATCTGGATTCTG  CCTAATAAAAAACATTTATTTTCATTGCTGCGTCGAGAGCTCGCTTTCTTGCTG  TCCAATTTCTATTAAAGGTTCCTTTGTTCCCTAAGTCCAACTACTAAACTGGGG  GATATTATGAAGGGCCTTGAGCATCTGGATTCTGCCTAATAAAAAACATTTATT  TTCATTGCTGCGTC |
| Poly-A | 126 | AAAAAAAAAAAAAAAAAAAAAAAAAAAAAAAAAAAAAAAAAAAAAAAAAAAAAA  AAAAAAATGCATAAAAAAAAAAAAAAAAAAAAAAAAAAAAAAAAAAAAAAAAAA  AAAAAAAAAAAAAAAAAA |
| **Genes** |  |  |
| **EGFP**  *(GenBank Accession #AAB02572.1)* | 720 | ATGGTGAGCAAGGGCGAGGAGCTGTTCACCGGGGTGGTGCCCATCCTGGTCGAG  CTGGACGGCGACGTAAACGGCCACAAGTTCAGCGTGTCCGGCGAGGGCGAGGGC  GATGCCACCTACGGCAAGCTGACCCTGAAGTTCATCTGCACCACCGGCAAGCTG  CCCGTGCCCTGGCCCACCCTCGTGACCACCCTGACCTACGGCGTGCAGTGCTTC  AGCCGCTACCCCGACCACATGAAGCAGCACGACTTCTTCAAGTCCGCCATGCCC  GAAGGCTACGTCCAGGAGCGCACCATCTTCTTCAAGGACGACGGCAACTACAAG  ACCCGCGCCGAGGTGAAGTTCGAGGGCGACACCCTGGTGAACCGCATCGAGCTG  AAGGGCATCGACTTCAAGGAGGACGGCAACATCCTGGGGCACAAGCTGGAGTAC  AACTACAACAGCCACAACGTCTATATCATGGCCGACAAGCAGAAGAACGGCATC  AAGGTGAACTTCAAGATCCGCCACAACATCGAGGACGGCAGCGTGCAGCTCGCC  GACCACTACCAGCAGAACACCCCCATCGGCGACGGCCCCGTGCTGCTGCCCGAC  AACCACTACCTGAGCACCCAGTCCGCCCTGAGCAAAGACCCCAACGAGAAGCGC  GATCACATGGTCCTGCTGGAGTTCGTGACCGCCGCCGGGATCACTCTCGGCATG  GACGAGCTGTACAAGTAA |
| **TA_EGFP**  *(Fused*  *K. pneumoniae transaminase gene (GenBank Accession #AF074934.1) and EGFP gene (GenBank Accession #AAB02572.1))* | 2002 | ATGAACAGCAACAAAGCGATGATGGCGCGCCGCAGCGATGCGGTGCCGCGCGGC  GTGGGCCAGATTCATCCGATTTTCGCGGAACGCGCGGAAAACTGCCGCGTGTGG  GATGTGGAAGGCCGCGAATATCTGGATTTTGCGGGCGGCATTGCGGTGCTGAAC  ACCGGCCATCTGCATCCGCAGGTGGTGGCGGCGGTGGAAGATCAGCTGAAGAAA  CTGAGCCATACCTGCTTTCAGGTGCTGGCGTATGAACCGTATCTGGCGCTGTGC  GAGAAAATGAACCAGAAAGTGCCGGGCGATTTTGCGAAGAAAACCCTGCTGGTG  ACCACCGGCAGCGAAGCGGTGGAAAACGCGGTGAAAATTGCGCGCGCGGCGACC  GGCCGCAGCGGCGCGATTGCGTTTACCGGCGCGGCGCATGGCCGCACCCATTAT  ACCCTGAGCCTGACCGGCAAAGTGAACCCGTATAGCGCGGGCATGGGCCTGATG  CCGGGCCATGTGTATCGCGCGCTGTATCCGTGCGCGCTGCATGGCGTGAGCGAT  GATGAAGCGATTGCGAGCATTCATCGCATTTTCAAGAACGATGCGGCGCCGGAA  GATATTGCGGCGATTATTATTGAACCGGTGCAGGGCGAAGGCGGCTTTTATGCG  GCGAGCCCGGCGTTTATGCAGCGCCTGCGCGCGCTGTGCGATGAACATGGCATT  ATGCTGATTGCGGATGAAGTGCAGAGCGGCGCGGGCCGCACCGGCACCCTGTTT  GCGATGGAACAGATGGGCGTGGCGGCGGATATTACCACCTTTGCGAAAAGCATT  GCGGGCGGCTTTCCGCTGGCGGGCGTGACCGGCCGCGCGGAAGTGATGGATGCG  ATTGCGCCGGGCGGCCTGGGCGGCACCTATGCGGGCAACCCGATTGCGTGCGCG  GCGGCGCTGGCGGTGCTGCAGATTTTCGAACAGGAAAACCTGCTGGAGAAAGCG  AACCAGCTGGGCGATACCCTGCGCCAGGGCCTGCTGGCGATTGCGGAAGATCAT  CCGGAAATTGGCGATGTGCGCGGCCTGGGCGCGATGATTGCGATTGAACTGTTT  GAAGAAGGCGATCATAGCCGCCCGAACGCGCGCCTGACCGCGGATATTGTGGCG  CGCGCGCGCGATAAAGGCCTGATTCTGCTGAGCTGCGGCCCGTATTATAACGTG  CTGCGCATTCTGGTGCCGCTGACCATTGAAGAAGCGCAGATTGAACAGGGCCTG  AAAATTATTGCGGATTGCTTTAGCGAAGCGAAACAGGCGCATGGTGAGCAAGGG  CGAGGAGCTGTTCACCGGGGTGGTGCCCATCCTGGTCGAGCTGGACGGCGACGT  AAACGGCCACAAGTTCAGCGTGTCCGGCGAGGGCGAGGGCGATGCCACCTACGG  CAAGCTGACCCTGAAGTTCATCTGCACCACCGGCAAGCTGCCCGTGCCCTGGCC  CACCCTCGTGACCACCCTGACCTACGGCGTGCAGTGCTTCAGCCGCTACCCCGA  CCACATGAAGCAGCACGACTTCTTCAAGTCCGCCATGCCCGAAGGCTACGTCCA  GGAGCGCACCATCTTCTTCAAGGACGACGGCAACTACAAGACCCGCGCCGAGGT  GAAGTTCGAGGGCGACACCCTGGTGAACCGCATCGAGCTGAAGGGCATCGACTT  CAAGGAGGACGGCAACATCCTGGGGCACAAGCTGGAGTACAACTACAACAGCCA  CAACGTCTATATCATGGCCGACAAGCAGAAGAACGGCATCAAGGTGAACTTCAA  GATCCGCCACAACATCGAGGACGGCAGCGTGCAGCTCGCCGACCACTACCAGCA  GAACACCCCCATCGGCGACGGCCCCGTGCTGCTGCCCGACAACCACTACCTGAG  CACCCAGTCCGCCCTGAGCAAAGACCCCAACGAGAAGCGCGATCACATGGTCCT  GCTGGAGTTCGTGACCGCCGCCGGGATCACTCTCGGCATGGACGAGCTGTACAA  GTAA |
| **T7 RNAP_**  **EGFP**  *(Fused*  *T7 RNA polymerase gene (GenBank Accession #NP_041960.1) and EGFP gene (GenBank Accession #AAB02572.1))* | 3370 | ATGAACACGATTAACATCGCTAAGAACGACTTCTCTGACATCGAACTGGCTGCT  ATCCCGTTCAACACTCTGGCTGACCATTACGGTGAGCGTTTAGCTCGCGAACAG  TTGGCCCTTGAGCATGAGTCTTACGAGATGGGTGAAGCACGCTTCCGCAAGATG  TTTGAGCGTCAACTTAAAGCTGGTGAGGTTGCGGATAACGCTGCCGCCAAGCCT  CTCATCACTACCCTACTCCCTAAGATGATTGCACGCATCAACGACTGGTTTGAG  GAAGTGAAAGCTAAGCGCGGCAAGCGCCCGACAGCCTTCCAGTTCCTGCAAGAA  ATCAAGCCGGAAGCCGTAGCGTACATCACCATTAAGACCACTCTGGCTTGCCTA  ACCAGTGCTGACAATACAACCGTTCAGGCTGTAGCAAGCGCAATCGGTCGGGCC  ATTGAGGACGAGGCTCGCTTCGGTCGTATCCGTGACCTTGAAGCTAAGCACTTC  AAGAAAAACGTTGAGGAACAACTCAACAAGCGCGTAGGGCACGTCTACAAGAAA  GCATTTATGCAAGTTGTCGAGGCTGACATGCTCTCTAAGGGTCTACTCGGTGGC  GAGGCGTGGTCTTCGTGGCATAAGGAAGACTCTATTCATGTAGGAGTACGCTGC  ATCGAGATGCTCATTGAGTCAACCGGAATGGTTAGCTTACACCGCCAAAATGCT  GGCGTAGTAGGTCAAGACTCTGAGACTATCGAACTCGCACCTGAATACGCTGAG  GCTATCGCAACCCGTGCAGGTGCGCTGGCTGGCATCTCTCCGATGTTCCAACCT  TGCGTAGTTCCTCCTAAGCCGTGGACTGGCATTACTGGTGGTGGCTATTGGGCT  AACGGTCGTCGTCCTCTGGCGCTGGTGCGTACTCACAGTAAGAAAGCACTGATG  CGCTACGAAGACGTTTACATGCCTGAGGTGTACAAAGCGATTAACATTGCGCAA  AACACCGCATGGAAAATCAACAAGAAAGTCCTAGCGGTCGCCAACGTAATCACC  AAGTGGAAGCATTGTCCGGTCGAGGACATCCCTGCGATTGAGCGTGAAGAACTC  CCGATGAAACCGGAAGACATCGACATGAATCCTGAGGCTCTCACCGCGTGGAAA  CGTGCTGCCGCTGCTGTGTACCGCAAGGACAGGGCTCGCAAGTCTCGCCGTATC  AGCCTTGAGTTCATGCTTGAGCAAGCCAATAAGTTTGCTAACCATAAGGCCATC  TGGTTCCCTTACAACATGGACTGGCGCGGTCGTGTTTACGCCGTGTCAATGTTC  AACCCGCAAGGTAACGATATGACCAAAGGACTGCTTACGCTGGCGAAAGGTAAA  CCAATCGGTAAGGAAGGTTACTACTGGCTGAAAATCCACGGTGCAAACTGTGCG  GGTGTCGATAAGGTTCCGTTCCCTGAGCGCATCAAGTTCATTGAGGAAAACCAC  GAGAACATCATGGCTTGCGCTAAGTCTCCACTGGAGAACACTTGGTGGGCTGAG  CAAGATTCTCCGTTCTGCTTCCTTGCGTTCTGCTTTGAGTACGCTGGGGTACAG  CACCACGGCCTGAGCTATAACTGCTCCCTTCCGCTGGCGTTTGACGGGTCTTGC  TCTGGCATCCAGCACTTCTCCGCGATGCTCCGAGATGAGGTAGGTGGTCGCGCG  GTTAACTTGCTTCCTAGTGAGACCGTTCAGGACATCTACGGGATTGTTGCTAAG  AAAGTCAACGAGATTCTACAAGCAGACGCAATCAATGGGACCGATAACGAAGTA  GTTACCGTGACCGATGAGAACACTGGTGAAATCTCTGAGAAAGTCAAGCTGGGC  ACTAAGGCACTGGCTGGTCAATGGCTGGCTCACGGTGTTACTCGCAGTGTGACT  AAGCGTTCAGTCATGACGCTGGCTTACGGGTCCAAAGAGTTCGGCTTCCGTCAA  CAAGTGCTGGAAGATACCATTCAGCCAGCTATTGATTCCGGCAAGGGTCCGATG  TTCACTCAGCCGAATCAGGCTGCTGGATACATGGCTAAGCTGATTTGGGAATCT  GTGAGCGTGACGGTGGTAGCTGCGGTTGAAGCAATGAACTGGCTTAAGTCTGCT  GCTAAGCTGCTGGCTGCTGAGGTCAAAGATAAGAAGACTGGAGAGATTCTTCGC  AAGCGTTGCGCTGTGCATTGGGTAACTCCTGATGGTTTCCCTGTGTGGCAGGAA  TACAAGAAGCCTATTCAGACGCGCTTGAACCTGATGTTCCTCGGTCAGTTCCGC  TTACAGCCTACCATTAACACCAACAAAGATAGCGAGATTGATGCACACAAACAG  GAGTCTGGTATCGCTCCTAACTTTGTACACAGCCAAGACGGTAGCCACCTTCGT  AAGACTGTAGTGTGGGCACACGAGAAGTACGGAATCGAATCTTTTGCACTGATT  CACGACTCCTTCGGTACCATTCCGGCTGACGCTGCGAACCTGTTCAAAGCAGTG  CGCGAAACTATGGTTGACACATATGAGTCTTGTGATGTACTGGCTGATTTCTAC  GACCAGTTCGCTGACCAGTTGCACGAGTCTCAATTGGACAAAATGCCAGCACTT  CCGGCTAAAGGTAACTTGAACCTCCGTGACATCTTAGAGTCGGACTTCGCGTTC  GCGCATGGTGAGCAAGGGCGAGGAGCTGTTCACCGGGGTGGTGCCCATCCTGGT  CGAGCTGGACGGCGACGTAAACGGCCACAAGTTCAGCGTGTCCGGCGAGGGCGA  GGGCGATGCCACCTACGGCAAGCTGACCCTGAAGTTCATCTGCACCACCGGCAA  GCTGCCCGTGCCCTGGCCCACCCTCGTGACCACCCTGACCTACGGCGTGCAGTG  CTTCAGCCGCTACCCCGACCACATGAAGCAGCACGACTTCTTCAAGTCCGCCAT  GCCCGAAGGCTACGTCCAGGAGCGCACCATCTTCTTCAAGGACGACGGCAACTA  CAAGACCCGCGCCGAGGTGAAGTTCGAGGGCGACACCCTGGTGAACCGCATCGA  GCTGAAGGGCATCGACTTCAAGGAGGACGGCAACATCCTGGGGCACAAGCTGGA  GTACAACTACAACAGCCACAACGTCTATATCATGGCCGACAAGCAGAAGAACGG  CATCAAGGTGAACTTCAAGATCCGCCACAACATCGAGGACGGCAGCGTGCAGCT  CGCCGACCACTACCAGCAGAACACCCCCATCGGCGACGGCCCCGTGCTGCTGCC  CGACAACCACTACCTGAGCACCCAGTCCGCCCTGAGCAAAGACCCCAACGAGAA  GCGCGATCACATGGTCCTGCTGGAGTTCGTGACCGCCGCCGGGATCACTCTCGG  CATGGACGAGCTGTACAAGTAA |

**Table S2.** Plasmid used in this study

| **Plasmids** | **Relevant characteristics** | **Source or references** |
| --- | --- | --- |
| pT7wt_EGFP | pUC57-Kan vector, wildtype T7 promoter, EGFP gene | ^23^ |
| pT7#4_EGFP | pUC57-Kan vector, T7#4 promoter, EGFP gene | ^42^ |
| pT7Max_EGFP | pUC57-Kan vector, T7Max promoter, EGFP gene | ^43^ |
| pT7c62_EGFP | pUC57-Kan vector, T7c62 promoter, EGFP gene | ^44^ |
| pT7Max_T7#4_EGFP | pUC57-Kan vector, T7Max_T7#4 promoter, EGFP gene | ^42,43^ |
| pT7c62_T7#4_EGFP | pUC57-Kan vector, T7c62_T7#4 promoter, EGFP gene | ^42,44^ |
| pT7DI_1_EGFP | pUC57-Kan vector, T7DI_1 promoter, EGFP gene | In this study |
| pT7DI_2_EGFP | pUC57-Kan vector, T7DI_2 promoter, EGFP gene | In this study |
| pT7DI_3_EGFP | pUC57-Kan vector, T7DI_3 promoter, EGFP gene | In this study |
| pT7DI_4_EGFP | pUC57-Kan vector, T7DI_4 promoter, EGFP gene | In this study |
| pT7DI_5_EGFP | pUC57-Kan vector, T7DI_5 promoter, EGFP gene | In this study |
| pT7DI_6_EGFP | pUC57-Kan vector, T7DI_6 promoter, EGFP gene | In this study |
| pT7DI_7_EGFP | pUC57-Kan vector, T7DI_7 promoter, EGFP gene | In this study |
| pT7DI_8_EGFP | pUC57-Kan vector, T7DI_8 promoter, EGFP gene | In this study |
| pT7DI_9_EGFP | pUC57-Kan vector, T7DI_9 promoter, EGFP gene | In this study |
| pT7DI_10_EGFP | pUC57-Kan vector, T7DI_10 promoter, EGFP gene | In this study |
| pT7DI_11_EGFP | pUC57-Kan vector, T7DI_11 promoter, EGFP gene | In this study |
| pET29A_TA | pET29A vector with *K. pneumoniae* transaminase gene | Lab collection |
| pET29A_T7 RNAP | pET29A vector with T7 RNA polymerase gene | Lab collection |
| pT7wt_TA_EGFP | Wildtype T7 promoter, fused *K. pneumoniae* transaminase and EGFP genes | In this study |
| pT7wt_T7 RNAP_EGFP | Wildtype T7 promoter, fused T7 RNA polymerase and EGFP genes | In this study |
| pT7DI_2_TA_EGFP | T7DI_2 promoter variant, fused *K. pneumoniae* transaminase and EGFP genes | In this study |
| pT7DI_2_T7 RNAP_EGFP | T7DI_2 promoter variant, fused T7 RNA polymerase and EGFP genes | In this study |
| pT7DI_7_TA_EGFP | T7DI_7 promoter variant, fused *K. pneumoniae* transaminase and EGFP genes | In this study |
| pT7DI_7_T7 RNAP_EGFP | T7DI_7 promoter variant, fused T7 RNA polymerase and EGFP genes | In this study |

**Table S3.** List of primers used in this study.

| **Primer name** | **Sequence (5’ 🡪 3’)** | **Template** |
| --- | --- | --- |
| 1. **Site-directed mutagenesis** | | |
| T7#4_FWD /  T7c62T7#4_FWD | ATAATACTCACTATTTGTTTTCGC | pT7wt_EGFP |
| T7Max_FWD | AATTCTAATACGACTCACTATAGGG | pT7wt_EGFP |
| T7c62_FWD | CGGAGACTCACTATTTGTTTTCGC | pT7wt_EGFP |
| T7MaxT7#4_FWD | AATTCTAATACGACTCACTATAGGG | pT7#4_EGFP |
| T7DI1_FWD | AATAAACTCACTATTTGTTTTCGC | pT7wt_EGFP |
| T7DI2_FWD | TAAAAACTCACTATTTGTTTTCGC | pT7wt_EGFP  pT7wt_TA_EGFP  pT7wt_T7 RNAP_EGFP |
| T7DI3_FWD | AATTAACTCACTATTTGTTTTCGC | pT7wt_EGFP |
| T7DI4_FWD | ATTAAACTCACTATTTGTTTTCGC | pT7wt_EGFP |
| T7DI5_FWD | TTAAAACTCACTATTTGTTTTCGC | pT7wt_EGFP |
| T7DI6_FWD | ATTTAACTCACTATTTGTTTTCGC | pT7wt_EGFP |
| T7DI7_FWD | TTTAAACTCACTATTTGTTTTCGC | pT7wt_EGFP  pT7wt_TA_EGFP  pT7wt_T7 RNAP_EGFP |
| T7DI8_FWD | ATTATACTCACTATTTGTTTTCGC | pT7wt_EGFP |
| T7DI9_FWD | ATATTACTCACTATTTGTTTTCGC | pT7wt_EGFP |
| T7DI10_FWD | TATATACTCACTATTTGTTTTCGC | pT7wt_EGFP |
| T7DI11_FWD | TTTTAACTCACTATTTGTTTTCGC | pT7wt_EGFP |
| T7#4/T7DI1-11_REV | CCCTATAGTGAGTCGTATTAC | pT7wt_EGFP |
| T7Max/  T7MaxT7#4_REV | GAATTCGATCTAGATGTATTCGCGAG | pT7wt_EGFP  pT7#4_EGFP |
| T7c62_REV | CGATTGTGAGTCGTATTACATCTAG | pT7wt_EGFP |
| T7c62T7#4_REV | CGCGATTGTGAGTCGTATTACATCTAG | pT7wt_EGFP |
| 1. **Colony PCR / Template production for IVT** | | |
| IVT0_FWD | TAATACGACTCACTATAGGGACTCACTATTTGTTTT | pT7wt_EGFP |
| IVT1_FWD | TAATACGACTCACTATAGGG | pT7#4_EGFP  pT7DI1_EGFP to pT7DI11_EGFP |
| IVT2_FWD | AATTCTAATACGACTCACTATAGGG | pT7Max_EGFP and pT7Max_T7#4_EGFP |
| IVT3_FWD | TAATACGACTCACAATCGCG | pT7c62_EGFP and  pT7c62_T7#4_EGFP |
| IVT_all_REV | TTTTTTTTTTTTTTTTTTTTTTTTTTTTTTTTTTTTTTTTTTTTTTTTTTTTTTTTTTTTATGCA |  |
| 1. **To create pT7wt_TA_EGFP (Gibson assembly)** | | |
| Vector_TA_FWD | AAACAGGCGCATGGTGAGCAAGGGCGAG | pT7wt_EGFP |
| Vector_TA_REV | TGCTGTTCATGGTGGCGACACTTTTTGC | pT7wt_EGFP |
| TA_FWD | TGTCGCCACCATGAACAGCAACAAAGCGATGATGG | pET29A_TA |
| TA_REV | TGCTCACCATGCGCCTGTTTCGCTTCGC | pET29A_TA |
| 1. **To create pT7wt_T7 RNAP_EGFP (Gibson assembly)** | | |
| Vector_T7 RNAP_FWD | GCGTTCGCGCATGGTGAGCAAGGGCGAG | pT7wt_EGFP |
| Vector_T7 RNAP_REV | TCGTGTTCATGGTGGCGACACTTTTTGC | pT7wt_EGFP |
| T7 RNAP_FWD | TGTCGCCACCATGAACACGATTAACATCGCTAAGAACGACTTC | pET29A_T7 RNAP |
| T7 RNAP_REV | TGCTCACCATGCGCGAACGCGAAGTCCG | pET29A_T7 RNAP |
| 1. **Standard sequencing primer** | | |
| M13 Forward (-43) | AGGGTTTTCCCAGTCACGACGTT | |

**Figure S1.** The agarose-gel electrophoresis analysis of IVT products (original gel images). IVT reactions were performed at 43^o^C for 2 h, based on the protocol in Rosa et al. (2022)^23^. The mRNA produced in the IVT is indicated by the 600 nt RNA band while the linear DNA template used in the reaction is represented by a 1.2 kb band. mRNA is produced in IVT reactions utilising T7 promoter variants, except for T7c62 and T7c62_T7#4, where no mRNA band is observed. Gels do not provide a normalised quantity of mRNA and cannot be used to assess the mRNA yield.


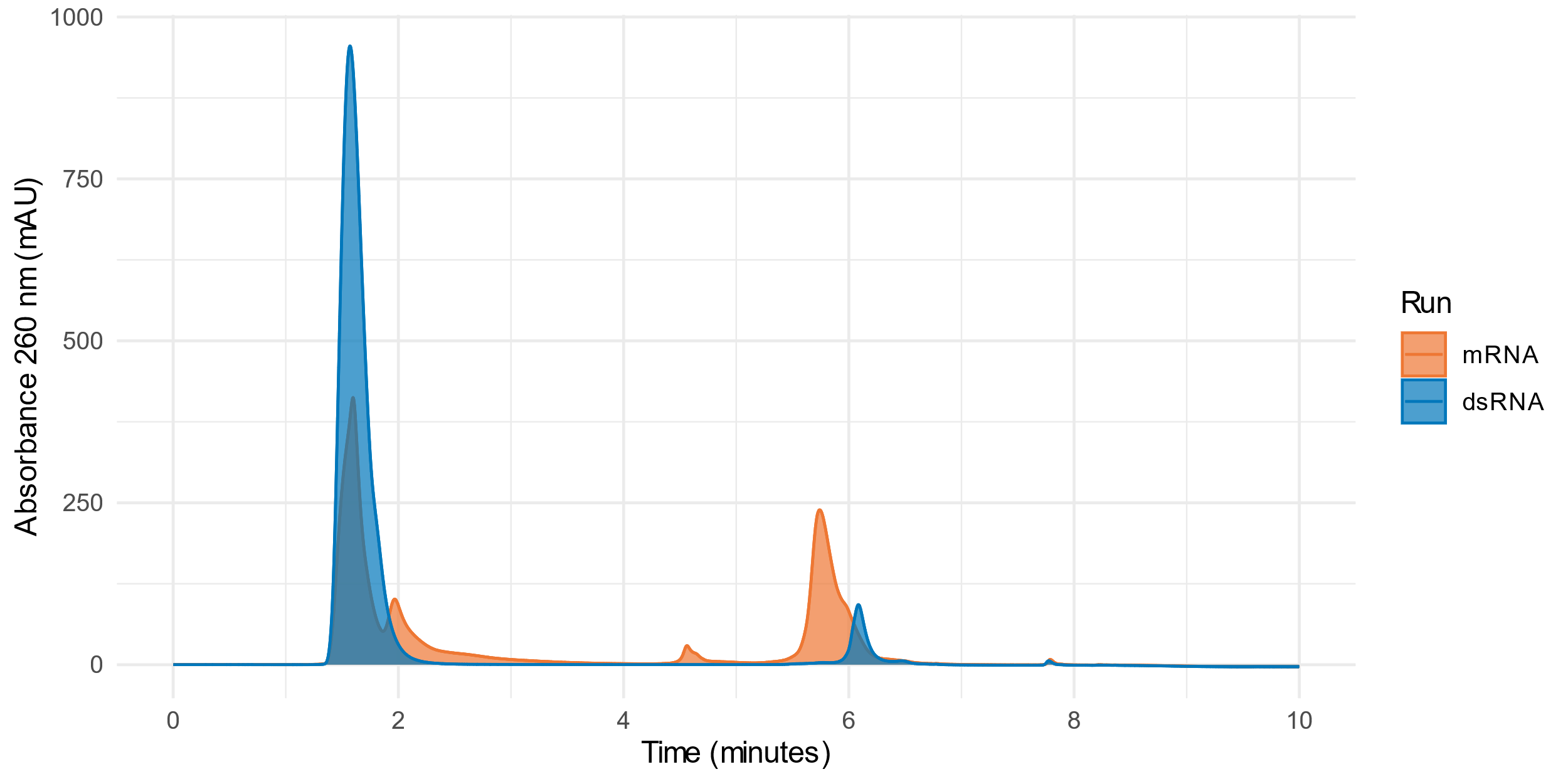


**Figure S2.** Chromatographic profiles of the same samples containing total mRNA (blue) and dsRNA (red). The dsRNA chromatogram was obtained after digestion with Rnase T1 as described in section 2.3.2. Peaks at 5.5 and 6.2 min are total mRNA and dsRNA, respectively.
